# Septic Arthritis of the Hip in a Young Athlete: A Diagnostic Delay Caused by Cognitive Bias

**DOI:** 10.7759/cureus.109741

**Published:** 2026-05-27

**Authors:** Kouichi Kajiwara, Shinsuke Sakoda, Kimiaki Kawano

**Affiliations:** 1 Department of Sports Medicine, Ashiya Central Hospital, Ashiya, JPN; 2 Department of Orthopaedic Surgery, Ashiya Central Hospital, Ashiya, JPN

**Keywords:** athlete, cognitive bias, diagnostic delay, hip, septic arthritis

## Abstract

Septic arthritis requires urgent diagnosis and treatment; however, acute hip pain in young athletes may be misinterpreted as a sports-related musculoskeletal condition, resulting in delayed recognition. We report a 21-year-old male rugby player who presented with rapidly worsening left hip pain after a match and difficulty bearing weight. Laboratory tests showed elevated inflammatory markers. Plain radiographs were normal, while magnetic resonance imaging demonstrated a hip joint effusion. Fluoroscopy-guided aspiration yielded approximately 11 mL of cloudy yellow synovial fluid, but antibiotic therapy was not initiated because a sports-related injury was initially suspected.

His symptoms and inflammatory markers temporarily improved with rest and anti-inflammatory medication, supporting the initial noninfectious impression. He was discharged but returned that evening with recurrent severe pain and an inability to walk. Repeat evaluation showed worsening inflammation, and contrast-enhanced computed tomography demonstrated persistent effusion with synovial enhancement. Synovial fluid culture subsequently grew methicillin-sensitive *Staphylococcus aureus *(MSSA). After MSSA was identified, targeted antibiotic therapy was started immediately, and arthroscopic irrigation and debridement were subsequently required. He successfully returned to competitive rugby 20 weeks postoperatively.

This case highlights how anchoring bias, together with initial clinical ambiguity and premature closure, can delay the diagnosis of septic arthritis. In patients with acute hip pain, joint effusion and elevated inflammatory markers should prompt prioritization of infection in the differential diagnosis. Cloudy synovial fluid should trigger immediate diagnostic reassessment, and a diagnostic timeout may be valuable when the clinical course is inconsistent with the initial impression.

## Introduction

Septic arthritis is an orthopedic emergency that requires prompt diagnosis and treatment because delayed management may result in irreversible cartilage destruction, osteomyelitis, systemic sepsis, and long-term functional impairment [1-4]. However, the condition is relatively uncommon in young, healthy adults and may be overlooked when classical systemic manifestations are absent or mild. In athletes, acute hip pain is often initially interpreted as a sports-related musculoskeletal problem, which may further delay consideration of infection.

The differential diagnosis of acute hip pain is broad and includes musculotendinous injury, labral pathology, transient synovitis, inflammatory arthritis, and occult fracture. In highly active patients, particularly athletes, symptoms are often first interpreted within a sports medicine framework [5]. Although this approach is frequently reasonable, it may inadvertently delay the consideration of infection.

Diagnostic error may result not only from missing clinical data but also from cognitive bias during clinical reasoning [6,7]. Anchoring on an initial impression and premature closure after an apparent early improvement may lead clinicians to maintain an incorrect working diagnosis despite contradictory findings.

We report a young rugby player with septic arthritis of the hip in whom a diagnostic delay occurred despite clinical and procedural findings suggestive of infection. This case highlights a reproducible decision-making pattern and emphasizes practical strategies to reduce similar delays in daily practice.

## Case presentation

A 21-year-old male rugby player presented with acute left hip pain. He had noticed mild discomfort before a match but remained able to play. The day after the match, his pain rapidly worsened, and he became unable to bear weight.

At initial presentation, his body temperature was 37.8°C. He demonstrated an antalgic gait due to severe left hip pain. Hip range of motion was limited by pain, with flexion restricted to approximately 70°. There was no clear traumatic event during play. He had no relevant medical history, no immunosuppression, recent intra-articular injection, or other known risk factors for joint infection. No recent skin abrasions, soft tissue infections, dental procedures, penetrating trauma, or other obvious sources of bacteremia were identified. Laboratory testing revealed a white blood cell count of 9,600/μL and a C-reactive protein level of 11 mg/dL (Table 1).

**Table 1:** Laboratory findings at initial presentation and readmission

Parameters	Initial Presentation	Readmission	Reference Range
White blood cell count (/μL)	9,600	10,800	3,300–8,600
C-reactive protein (mg/dL)	11	13	<0.3

**Figure 1:**
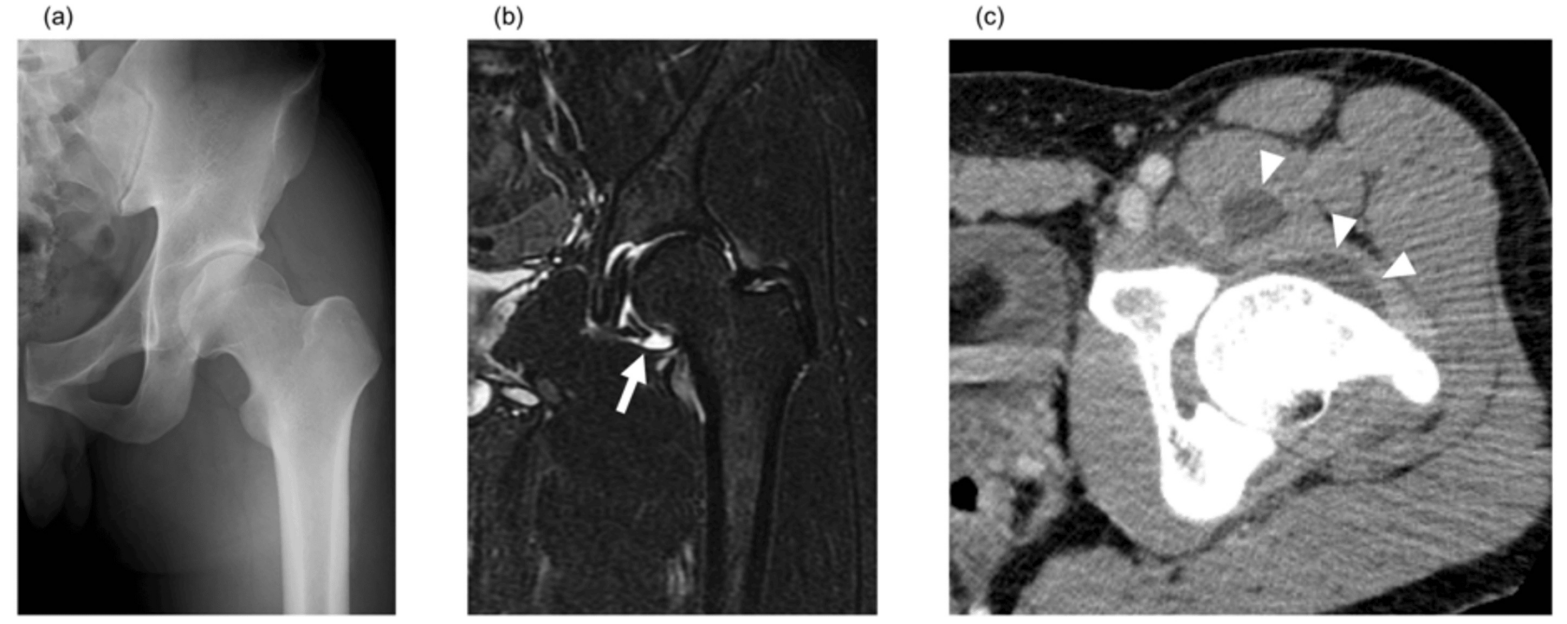
Imaging findings of the left hip during the clinical course (a) Anteroposterior plain radiograph obtained at initial presentation showing preserved joint space without obvious osseous abnormality. (b) Coronal STIR magnetic resonance image obtained at initial presentation demonstrating a left hip joint effusion (arrow). (c) Contrast-enhanced computed tomography obtained after symptom recurrence showing persistent joint effusion, capsular distension, increased synovial enhancement, and fluid extension toward the iliopsoas bursa (arrowheads). STIR: Short tau inversion recovery.

Plain radiographs showed no osseous abnormality. Magnetic resonance imaging demonstrated a left hip joint effusion (Figure 1, Panels a and b). As symptom onset followed athletic activity and imaging showed no structural bone injury, the initial working diagnosis was a sports-related musculoskeletal injury.

Given the severity of pain relative to the imaging findings, he was admitted for further evaluation. Fluoroscopy-guided hip aspiration yielded approximately 11 mL of cloudy yellow synovial fluid. Despite this finding, septic arthritis was not considered the leading diagnosis at that time, and antibiotic therapy was not initiated.

During hospitalization, his pain improved rapidly with rest and nonsteroidal anti-inflammatory medication, and inflammatory markers showed a downward trend. Based on this transient response, a noninfectious inflammatory condition was favored, and he was discharged without antibiotics.

However, later the same day, severe hip pain recurred without an apparent trigger, and he again became unable to walk. On readmission, the white blood cell count had increased to 10,800/μL and the C-reactive protein level to 13 mg/dL. Contrast-enhanced computed tomography demonstrated persistent joint effusion, capsular distension, synovial enhancement, and fluid extension toward the iliopsoas bursa (Figure 1, Panel c). Ultrasonography also confirmed persistent hip joint effusion during readmission.

**Figure 2:**
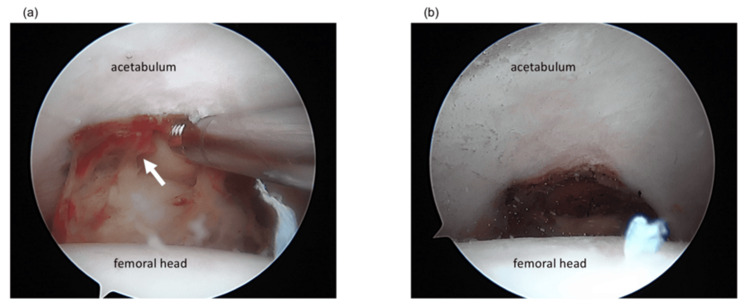
Arthroscopic findings of the left hip (a) Hyperemic synovium and intra-articular debris on the acetabular side (arrow). (b) Improved synovitis and a clear intra-articular view after irrigation and debridement.

At that time, septic arthritis of the hip was strongly suspected. Synovial fluid culture obtained during the initial admission subsequently grew methicillin-sensitive *Staphylococcus aureus* (MSSA). After MSSA was identified, antibiotic therapy with levofloxacin, rifampicin, and clindamycin was started immediately. As clinical improvement was insufficient, arthroscopic irrigation and debridement were performed. Intraoperatively, hyperemic synovium and intra-articular debris were observed. After lavage and debridement, the intra-articular view became clear (Figure 2).

Postoperatively, both symptoms and inflammatory markers improved steadily. Clindamycin was discontinued after normalization of inflammatory markers, followed by gradual de-escalation of antibiotic therapy to levofloxacin monotherapy. He completed a staged rehabilitation program and returned to competitive rugby 20 weeks after surgery. Follow-up radiographs obtained approximately nine months postoperatively showed no apparent osteoarthritic changes, and he remained asymptomatic during sports participation.

## Discussion

The diagnostic delay in this case was not caused by a single overlooked finding but rather by a sequence of decisions shaped by cognitive bias during the diagnostic process. At the initial presentation, the history of hip pain following a rugby match favored a sports-related musculoskeletal explanation. Infection was therefore not prioritized in the differential diagnosis, and subsequent clinical data were interpreted within that initial framework.

The most important missed opportunity occurred when hip aspiration yielded cloudy yellow synovial fluid. Cloudy synovial fluid is highly concerning for septic arthritis, and infection should be prioritized in patients presenting with acute monoarticular pain, particularly when accompanied by joint effusion and elevated inflammatory markers [2,8-10]. In this case, however, the context of a young athlete with symptom onset following exercise likely reinforced a noninfectious interpretation and reduced the perceived significance of this finding.

Symptoms and inflammatory markers temporarily improved after admission with rest and anti-inflammatory treatment, which further strengthened the initial working diagnosis. This pattern can be explained by anchoring bias and premature closure, in which clinicians remain attached to an early impression and prematurely terminate diagnostic reconsideration [6,7].

Although septic arthritis is often expected to show progressive deterioration, infection cannot be safely excluded based on early transient improvement alone [11,12]. In the present case, this temporary response was interpreted as evidence against infection and contributed to delayed reassessment. Synovial fluid cell count and Gram stain results were unavailable because these tests were not routinely performed at the time of treatment.

The central lesson of this case is that diagnostic delay due to cognitive bias represents a reproducible clinical pattern rather than an isolated error. Similar reasoning failures may occur whenever a plausible mechanical explanation is available and discordant findings are given insufficient weight. At the same time, initial clinical ambiguity and transient symptomatic improvement also contributed to the diagnostic challenge in this case.

Several practical safeguards may help prevent similar delays. When joint effusion and elevated inflammatory markers are present, septic arthritis should remain a priority diagnosis [2,9,10]. When cloudy synovial fluid is obtained, the working diagnosis should be immediately reconsidered [8-10]. In addition, when the subsequent clinical course is inconsistent with the initial hypothesis, a structured diagnostic timeout may help reopen the differential diagnosis and reduce fixation on an incorrect diagnosis [6,7].

## Conclusions

This case demonstrates how cognitive bias, together with initial clinical ambiguity, can delay the diagnosis of septic arthritis in a young athlete with acute hip pain. Anchoring on a sports-related mechanism and premature closure after transient improvement contributed to the underrecognition of findings suggestive of infection.

When joint effusion and elevated inflammatory markers are present, septic arthritis should remain a priority diagnosis regardless of patient age or athletic status. Cloudy synovial fluid warrants immediate reassessment of the working diagnosis, and a diagnostic timeout should be considered whenever the subsequent clinical course is inconsistent with the initial assumptions.
